# The PD-1:PD-L1 axis in Inflammatory Arthritis

**DOI:** 10.1186/s41927-020-00171-2

**Published:** 2021-01-11

**Authors:** Mary Canavan, Achilleas Floudas, Douglas J. Veale, Ursula Fearon

**Affiliations:** 1 Department of Molecular Rheumatology, Trinity Biomedical Sciences Institute, Trinity College Dublin, Dublin 2, Ireland; 2 EULAR Centre of Excellence, Centre for Arthritis & Rheumatic Diseases, St. Vincent’s University Hospital, University College Dublin, Dublin, Ireland

**Keywords:** Checkpoint inhibitors, PD-1, Rheumatoid arthritis, Psoriatic arthritis, Adverse events

## Abstract

The activation of antigen specific T cells during an immune response is a tightly regulated process at the level of both costimulatory and coinhibitory receptors. One such coinhibitory receptor or checkpoint inhibitor which has received much attention in the field of oncology is the programmed cell death protein 1 (PD-1). Blockade of PD-1 or its ligand PD-L1 has proven successful in the treatment of a wide variety of cancers, therefore highlighting an important role for this pathway in anti-tumour immune responses. However, a caveat of PD-1 therapy and boosting anti-tumour immune responses is the development of self-reactive T cells which can lead to the induction of various autoimmune or inflammatory diseases, referred to as immune- related adverse events (irAEs). The emergence of rheumatological irAEs such as Inflammatory Arthritis (IA) in recent years has highlighted the importance of PD-1 in maintaining self-tolerance. Furthermore, the emergence of rheumatology related irAEs raises an important question as to how defects in this pathway can contribute to spontaneous rheumatological disease. In this review, we describe the biological distribution, function and regulation of the PD-1 pathway, its potential role in IA and irAE related IA.

## Background

It is widely appreciated that costimulation of T cells via antigen presenting cells (APC) is an essential step in boosting the immune system and inducing antigen specific T cell responses. Costimulatory receptors such as CD80 and CD86, expressed on the surface of APC, bind the CD28 molecule on T cells, driving proliferation and cytokine production. Following resolution of infection, coinhibitory receptors become upregulated to prevent destruction of host tissues and to restore homeostasis [1]. Therefore, both positive and negative signals are required to regulate T cell function. Programmed cell death protein 1 (PD-1) acts as a negative regulator or immune checkpoint inhibitor (ICI) of T cell responses. The PD-1 axis acts as an essential pathway to restore tolerance and prevent the accumulation of self-reactive T cells [2]. This review centres on the PD-1 pathway, its long-appreciated role in tolerance and more recent advances on its role in autoimmune diseases and cellular metabolism. Furthermore, we will examine the regulation of this checkpoint inhibitor in Inflammatory Arthritis (IA), while discussing the emergence of IA in ICI treated patients in the cancer setting.

### PD-1 expression, signalling and regulation

PD-1 (also known as CD279) is a 55 kDa type I transmembrane protein belonging to the CD28 superfamily of immunoreceptors. It is primarily expressed on immune cells such as T, B and NK cells, in addition to monocytes, macrophages and dendritic cells (DC). However, to date, studies exploring PD-1 signalling and its functions have been performed predominantly on T cells. While PD-1 is usually absent on naïve T cells, its expression following antigen engagement of the T cell receptor (TCR), is significantly upregulated [3, 4]. Once this antigen is sufficiently cleared, the PD-1 receptor is then subsequently downregulated. However, during periods of chronic infection or indeed cancer, where elimination of antigen in its entirety is inefficient, PD-1 expression remains high. Previous studies have highlighted that in these settings of chronic antigen encounter (i.e. chronic infection and cancer), T cells become exhausted rendering them unable to secrete cytokines, proliferate or perform their effector functions [5, 6]. In this setting, high expression of PD-1 is a hallmark of exhausted T cells [7].

**Fig. 1:**
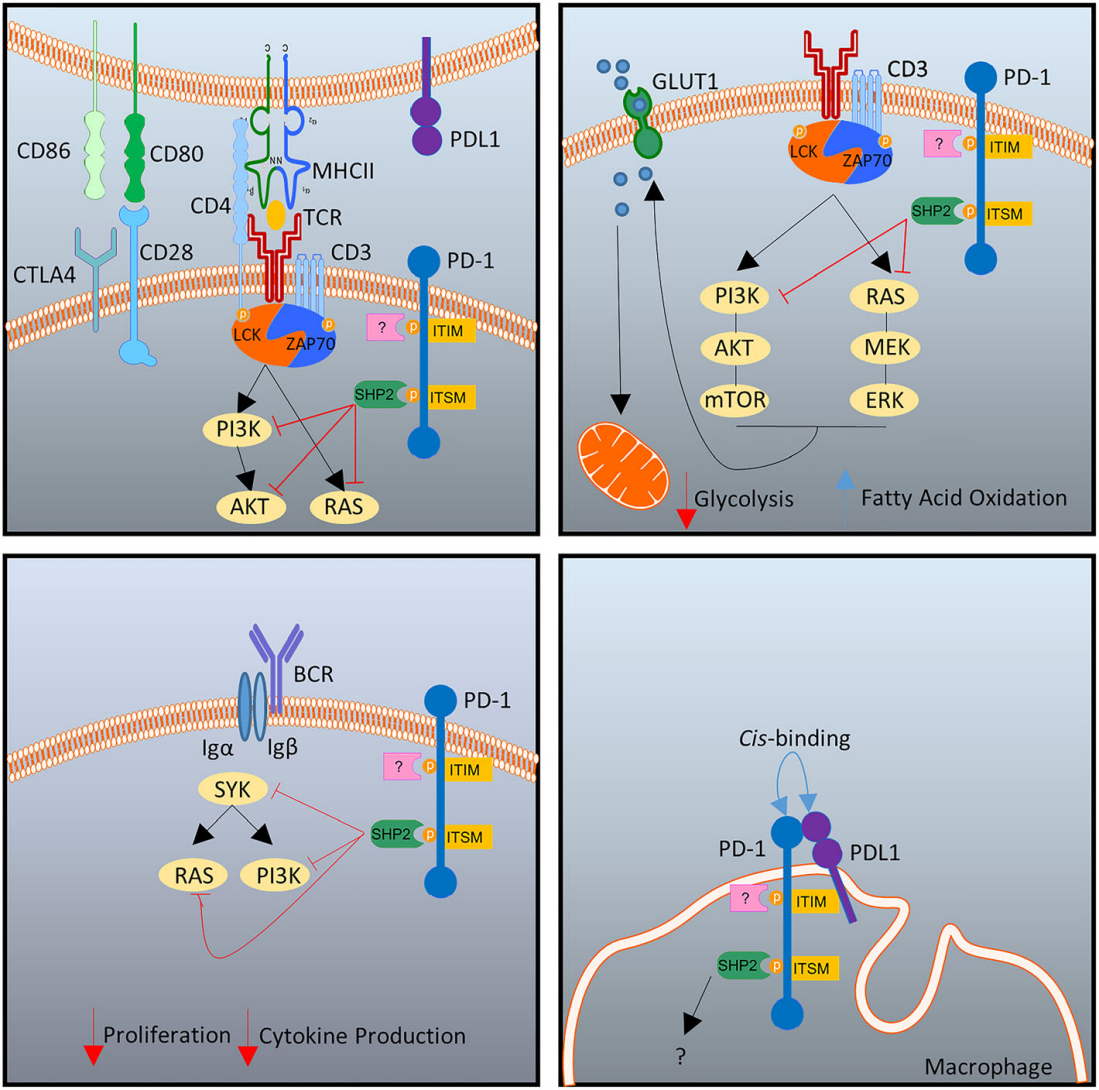
Mechanisms of action of PD-1. During immunological synapse formation, ligation of PD-1 with PD-L1 leads to the recruitment of SHP-2 at the ITSM site of PD-1 and subsequent dephosphorylation of PI3K, AKT and RAS dampening down TCR mediated signalling, top left panel. Due to inhibition of PI3K and RAS, PD-1 can change the metabolic profile of the T cell by limiting GLUT-1 expression and mitochondrial availability of glucose, favouring fatty acid oxidation over glycolysis, top right panel. There is a paucity of data on the role of B cell PD-1, however it has been suggested that PD-1 leads to inhibition of SYK resulting in reduced B cell proliferation and cytokine production following B cell receptor (BCR) mediated activation, bottom left panel. A recently proposed mechanism of action for macrophage PD-1 expression is binding to PDL-1 in cis and therefore limiting available PD-L1 for ligation with T cell PD-1, bottom right panel

PD-1 interacts with two known ligands, PD-L1, also known as B7-H1 or CD274 and PD-L2, also known as B7-DC or CD273. PD-L1 is expressed on a wide variety of cell types including T and B cells, macrophages, DC and mast cells, in addition to being expressed in tissues such as heart, lung, kidney and liver. The expression of PD-L2 however, appears to be more restricted, being detected only on APCs such as macrophages and DC, where its expression is regulated by cytokines including IFN-γ, GMCSF and IL-4 [8, 9]. The expression of PD-L1 is regulated in response to numerous cytokines such as type I and type II interferons, IL-10, IL-17, IL-6, IL-4, IL-1β and IL-27 [10–13]. In addition to regulation by the aforementioned cytokines, PD-L1 expression is also induced by a number of signalling pathways and pattern recognition receptors. Specifically, NFκB, MAPK, HIF and STAT3 have all been implicated in PD-L1 induction [12, 14–16]. Moreover, following engagement of the pattern recognition receptors TLR4 and TLR3, PD-L1 expression is also regulated [17, 18]. PD-1 preferentially ligates to PD-L1 over PD-L2 for reasons not fully understood at present. While PD-1:PD-L2 has a higher binding affinity compared to PD-1:PD-L1, PD-L2 is generally expressed at lower levels than PD-L1, thus favouring a PD-1:PD-L1 partnership [19]. Following ligation with PD-L1, activated PD-1 can antagonize the TCR signal transduction pathway through several mechanisms. Firstly, PD-1 is expressed as a monomer on the cell surface, where it consists of an extracellular immunoglobulin-like binding domain, a transmembrane region and a cytoplasmic domain which contains two tyrosine motifs – an inhibitory motif (ITIM) and an immunoreceptor switch motif (ITSM) [20]. A conformational change in the PD-1 receptor is induced following ligation to PD-L1, thus resulting in the phosphorylation of both tyrosine motifs - ITIM and ITSM by Src family kinases [21–23]. Subsequently, SHP-1 and SHP-2 tyrosine kinases are recruited, resulting in the dephosphorylation of several kinases which can inhibit both TCR and CD28 mediated signals leading to a reduction in T cell proliferation, survival and cytokine production [24–28] (Fig. 1, top left panel). Bidirectional signalling following PD-L1 ligation may also occur, however, studies into this area are limited, in part due to the lack of evidence identifying intracellular signal transduction pathways downstream of PD-L1 activation. Until recently, no identifiable cytoplasmic signal transductions motifs were identified on PD-L1. However, a recent study by Gato-Canas et al. elegantly identified functional regulatory signal motifs within the intracytoplasmic domain of PD-L1 which may be responsible for PD-L1 reverse signalling [29]. While the specific signalling pathways induced by PD-L1 reverse signalling is unclear, effects of PD-L1 activation on APC have been reported. DC treated with soluble PD-1 (sPD-1) exhibit decreased maturation profiles and increased production of IL-10 [30]. Moreover, within the context of inflammatory disease, autoantibodies to PD-L1 have been identified within the serum of Rheumatoid Arthritis (RA) patients which can facilitate bidirectional PD-L1 signalling on T cells. Specifically, PD-L1 activation on T cells induced increased secretion of IL-10, in addition to a small increase in IFN-γ secretion [31].

The regulation of PD-1 expression on T cells is dependent on the context of antigen exposure, whereby acute or chronic infection (or the persistence of tumour antigen in the cancer setting) dictates the transcriptional pathways used to regulate its expression. Following T cell activation through the TCR, the inducible transcription factors, nuclear factor of activated T cells 1c (NFAT1c) and Notch are responsible for the initial expression of PD-1 in both CD4^+^and CD8^+^ T cells [32, 33]. However, these transcription factors are dispensable for the maintenance of PD-1 expression during chronic infection. The transcription factor forkhead box protein O1 (FoxO1) has been identified as a key regulator of PD-1 expression during chronic inflammation, whereby high expression of FoxO1 inhibits T-bet expression [34]. Given that T-bet is a previously identified repressor of PD-1, inhibition of T-bet via FoxO1 leads to the accumulation of PD-1 on T cells [35]. NFAT1c has also been implicated in driving PD-1 expression in B cells [36]. Moreover, in addition to T and B cells, the regulation of PD-1 has also been examined in macrophages, whereby NFκB signalling mediates PD-1 expression following TLR stimulation [36]. While PD-1 is mainly upregulated on T cells in response to antigen via TCR signalling, antigen independent PD-1 regulation has also been described. Kinter et al. demonstrated an induction in PD-1 expression on peripheral blood T cells in response to the common gamma chain cytokines IL-2, IL-7, IL-15, and IL-21 [37]. Furthermore, anti-inflammatory cytokines such as IL-10 and TGF-β have also been shown to regulate PD-1 expression on T cells [38, 39].

### PD-1 and tolerance

The first evidence for the key role of PD-1 in regulating T cell tolerance was discovered following the generation of PD-1^−/−^ mice. PD-1 deficient T cells were hyperproliferative and upon introduction of the *lpr* mutation, PD-1^−/−^ mice developed more severe lupus like disease compared to PD-1^+/+^ littermates [40]. PD-1 is expressed by CD4^−^CD8^−^ double negative (DN) T cells during thymic development [41]. In the absence of PD-1, there is a reported increase in CD4^+^CD8^+^ double positive (DP) T cells, providing evidence for a PD-1 mediated augmentation of positive selection in the thymus. The effect of PD-1 on thymic T cell development is not monospecific either, since PD-1 exerts a negative effect on the selection of the TCR β chain that can lead to bias of the T cell repertoire [41]. In a more recent study, Jiang et al. demonstrated that PD-1 limits the escape of high affinity autoreactive CD4^+^ T cells from the thymus. The authors tracked individual autoreactive T cell clones transferred to lymphopenic recipient mice and observed that PD-1 expression can limit the expansion of autoreactive T cells in specific tissues, therefore creating a link between PD-1 deprivation and autoimmunity [42]. Upon antigen recognition, T cells become arrested by APC, preventing their migration away from the immunological synapse thus enabling appropriate T cell-APC signalling to occur. Following T cell activation and cytokine production, T cells can subsequently regain their motility and minimise cytokine production. During these T cell-APC interactions, T cell PD-1 expression is upregulated and the T cell fine tunes the time spent at this immunological synapse. In the absence of PD-1, the length of the T cell-APC interaction increases, therefore resulting in increased pro-inflammatory cytokine production [43]. The effect of PD-1 on T cell velocity and motility is specific to PD-1 and potentially dependent on antigen availability, with CTLA-4 (Cytotoxic T lymphocyte antigen-4) not exhibiting a similar effect [44]. Interestingly, PD-1:PD-L1 and PD-1:PD-L2 interactions are potentially not equal in their capacity to maintain peripheral T cell tolerance. In a mouse model of diabetes, PD-L1 blockade inhibited T cell tolerance, while PD-L2 blockade did not [45]. PD-1 is rapidly upregulated following TCR engagement and can effectively block T cell transition from naïve to effector T cell, however anergic T cells have a similar PD-1 expression level to effector T cells and do not become invigorated following PD-1 blockade [46, 47]. PD-1 blockade can also affect T cell chemokine expression by enhancing effector T cell CXCR3 expression, therefore inducing migration to the target tissue [47].

In addition to the capacity of the PD-1:PD-L1 axis to regulate effector T cell responses, emerging evidence suggests a key role for PD-1 in the development and function of T regulatory cells (Treg). Tregs can regulate the immune response by utilising contact dependent and contact independent mechanisms of action. These cells are characterised by high expression of the transcription factor Foxp3 and the IL-2 receptor α chain (CD25) [48]. Treg cells perform immunosuppressive functions in a contact dependent manner through CTLA-4. CTLA-4 is constitutively expressed by Treg cells and binds with high affinity to CD80 and CD86 leading to decreased availability of CD28 mediated costimulation and T cell activation. In addition to CTLA-4, PD-1 ligation has also been reported to maintain expression of Foxp3, with PD-L1 being essential for the in vitro induction of Treg cells through downregulation of the mTOR pathway [49]. PD-1 engagement of Th1 cells in vivo has been shown to induce plasticity and lasting conversion of Th1 cells to Tregs, which is dependent on intact downstream signalling of PD-1 [50]. The immunosuppressive capacity of Tregs is not restricted to the T cell compartment, with Treg cells being able to suppress activation and autoantibody production specifically of autoreactive PD-1^+^ B cells in a PD-1:PD-L1dependant manner [51].

The effect of PD-1 on immune tolerance is not restricted to T cells, some B cells, macrophages and DC also express PD-1, however the role(s) of PD-1 expression on these cells appear pleiotropic and remain poorly understood. Recent murine studies indicate that tumour associated macrophages (TAM) maintain high expression of PD-1 that correlate negatively with their capacity to phagocytose tumour cells [52]. PD-1 expressing TAM maintain an M2-like phenotype with high expression of CD206. These cells are potentially infiltrating macrophages that have originated from the bone marrow [52]. Similar to macrophages, PD-1 expressing human DC have been shown to limit anti-tumour immunity by suppressing CD8^+^ T cell IL-2 and IFN-γ production [53]. Engagement of PD-1 on macrophages and DC can have pleiotropic effects leading to enhanced rather than suppressed immune responses. This is based on the observation that PD-1 can bind PD-L1 expressed by the same cell in *cis,* therefore, limiting the availability of PD-L1 on the APC [54] (Fig. 1, bottom right panel). This cis interaction of PD-1:PD-L1 is an additional functional mechanism used within the PD-1 pathway, in addition to the more common method of trans activation reported in the previous section above. A small population of peripheral blood B cells also express PD-1. While there is a paucity of evidence, initial studies show that B cell PD-1 expression is a result of B cell receptor (BCR) engagement and dampens down BCR mediated signalling by recruitment of SHP-2 [55] (Fig. 1, bottom left panel). PD-1 expressing B cells have also been shown to accumulate in thyroid tumours, and while they do not express higher IL-10 than PD-1 negative B cells, they were able to suppress T cell responses in a PD-1:PD-L1 dependent manner [56].

### PD-1 and metabolism

It is now widely appreciated that the activation, proliferation and effector functions of immune cells are intrinsically linked to cellular metabolism. Cellular bioenergetics are adapted towards the specific functional requirements of the cell, and in addition to meeting cellular ATP demands, also provide biosynthetic intermediates. In the context of autoimmune disease, specifically Rheumatoid Arthritis (RA) and Psoriatic Arthritis (PsA), we and others have shown that altered cellular bioenergetics due to mitochondrial dysfunction, oxidative stress and hypoxia, drive pro-inflammatory processes in the synovial tissue in inflamed diarthrodial joints. Moreover, nutrient supply and signalling pathways such as HIF-1α, NFκB, Notch-1 and JAK-STAT have also been shown to mediate metabolic changes in RA [57–59]. While principally the main role of the PD-1 pathway is to act as an inhibitory receptor for immune responses, reports are now emerging of a potential role for the PD-1:PD-L1 axis in metabolism. While activated T cells undergo rapid metabolic reprogramming to glycolysis to support their proliferation and effector functions, studies have shown that following PD-1 ligation, activated T cells no longer utilise glycolysis, glutaminolysis or metabolism of branched-chain amino acids, but instead use fatty acid oxidation (FAO) to generate energy [60]. Moreover, PD-1 ligated T cells display a significant decrease in their expression of the glucose transporter Glut1, therefore impairing their ability to take up glucose (Fig. 1, top right panel). Patsoukis et al. demonstrated a reduction in the extracellular acidification rate (ECAR) and oxygen consumption rate (OCR) in PD-1 stimulated T cells, indicating an overall reduction in the ability of the cells to generate energy when glucose is the energy source. Furthermore, PD-1 activated T cells have increased spare respiratory capacities (SRC), thus enabling continued ATP production under increased cellular stress. Moreover, additional studies have demonstrated a reduction in cellular glycolysis following PD-1 ligation in vivo*.* Bengsch et al. explored the role of PD-1 signalling in exhausted CD8^+^ T cells during chronic lymphocytic choriomeningitis virus (LCMV) infection. They demonstrated that exhausted T cells had reduced oxidative phosphorylation, decreased glucose uptake and reduced glycolysis, mediated in part, by the PD-1 pathway [61]. Oganda et al. recently suggested the mitochondria itself may be the main target of the PD-1 inhibitory pathway. The authors reported a reduction in mitochondrial polarization and a decrease in several genes involved in mitochondrial structure and function in CD8^+^ T cells following PD-1 stimulation. Importantly, this led to a decrease in the number and length of mitochondrial cristae, suggesting an impairment in glucose metabolism may be in part due to mitochondrial dysfunction [62].

### The PD-1 pathway in inflammatory arthritis

The PD-1:PD-L1 axis has been examined in Inflammatory Arthritis in an effort to understand how negative regulators function in the context of chronic inflammation. The expression of PD-1 and its ligand PD-L1 are upregulated in the RA synovium within lymphoid aggregates of the sub lining layer. We previously examined the PD-1:PD-L1 pathway within RA disease progression and subsequently demonstrated a significant increase in synovial PD-1 expression in early and established RA compared to both healthy control and Osteoarthritis (OA) synovial tissue. Furthermore, the expression of the PD-1 ligands, PD-L1 and PD-L2 were significantly increased in both early and established RA as well as arthralgia and undifferentiated IA [63]. Previous studies have also confirmed that histological expression of PD-1 correlates with the degree of synovial inflammation [64]. sPD-1 has also been detected in RA and PsA synovial fluid and serum, while being absent in OA [65]. sPD-1 levels are also increased in the serum of ACPA-positive but not ACPA-negative RA patients [63]. Interestingly, PD-1 has also been detected within extracellular vesicles (EV) in RA plasma and synovial fluid [66]. We and others have identified PD-1 expression on CD4^+^ and CD8^+^ T cells within the RA and PsA synovium [63, 65, 67, 68]. The frequency of PD-1 expressing CD4^+^ and CD8^+^ T cells is also significantly elevated in RA synovial fluid compared to RA peripheral blood [69]. Here, the expression of PD-1 on these synovial fluid T cells correlates with disease activity [69]. Interestingly, PD-1 expression is absent on OA synovial T cells, suggesting its expression may be involved in IA pathogenesis rather than merely a consequence of inflammation [70]. Within the periphery, a decrease in the percentage of circulating CD4^+^ and CD8^+^ PD-1^+^ cells in the blood of PsA and RA patients, respectively, has also been reported [71, 72]. In the context of myeloid cells, PD-L1 is expressed on synovial fluid CD1c^+^ dendritic cells [68], in addition to synovial fluid macrophages [67]. Moreover, PD-1−/− mice develop more severe arthritis, while PDL-1.Fc treatment can inhibit the development of collagen induced arthritis (CIA) [64] and polymorphisms in PD-1 are associated with increased risk of developing RA [73]. To date, no previous studies have directly examined the effect of biologic or disease-modifying antirheumatic drugs (DMARD) treatment on the PD-1:PD-L1 axis in IA. However, the role that the therapeutically targeted cytokines, TNFα and IL-6 have on the PD-1 pathway has previously been examined by Bommarito et al. This study demonstrated that while PD-L1 activation in healthy CD4^+^ T cells results in decreased T cell proliferation, this effect is abrogated in the presence of the pro-inflammatory cytokines TNFα, IL-6 or IL-1β. Moreover, upon addition of the anti-TNFα drug adalimumab, anti-IL-6R drug tocilizumab or anti-IL-1β mAb, these cytokine-mediated effects are reversed and PD-L1 mediated T cell suppression is restored [65]. Furthermore, we previously demonstrated that the gene signature induced by the antagonistic anti-PD-1 antibody, nivolumab (representing genes enriched as a result of inhibition on the PD-1:PD-L1 axis), is enriched in early and established RA patients. Upon examination of the effect of treatment on this enrichment, we noted a reduction in this enrichment signature in early RA synovial tissue following DMARD treatment, suggesting that DMARD treatment may normalise the PD-1 pathway in RA [63].

**Fig. 2:**
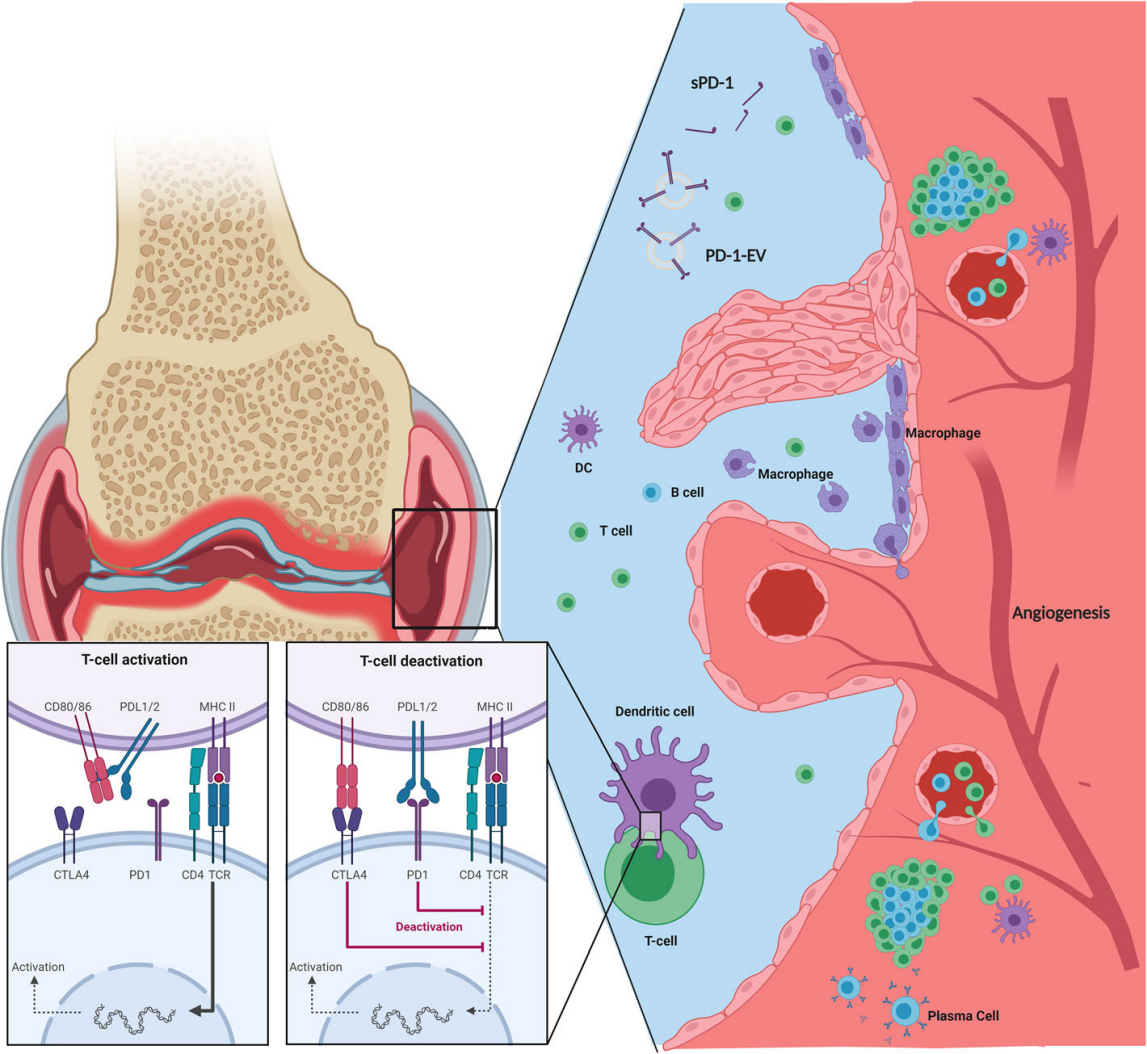
The PD-1 pathway in Inflammatory Arthritis. Despite increased expression of PD-1 by CD4+ and CD8+ T cells in the synovial tissue of RA patients, increased sPD-1 and PD-1 carrying EV could inhibit PD-1 mediated T cell suppression. Additionally, availability of PD-L1 by synovial DC could be limited due to increased expression of CD80 and the binding of CD80 to PD-L1 in cis, therefore, reducing the functionally available PD-L1

While the overexpression of the PD-1 pathway in RA may appear contradictory given the persistence of activated and proliferating T cells within the synovium, Wan et al. also provide evidence to justify this corollary. High levels of sPD-1 were identified in RA synovial fluid, which can antagonise the function of PD-1^+^ T cells. Studies by Bommarito et al. confirmed these findings in RA and subsequently reported similar results in PsA, whereby increased sPD-1 in PsA SF may counteract PD-1 mediated T cell suppression [65]. Furthermore, upon examination of the function of PD-1^+^ T cells in RA, Raptopoulou et al. demonstrated a less pronounced PD-1 mediated reduction in T cell proliferation in RA synovial T cells compared to peripheral blood, suggestive that T cells within the synovium may be more resistant to PD-1 mediated suppression [64]. Moreover, we have previously reported a lack of PD-L1 positive cells in the RA synovium, and as mentioned above, a decrease in the PD-1 pathway gene signature in RA (i.e. enrichment of the nivolumab gene signature). Taken together, our data and that of others suggest that although PD-1 is present, the pathway may be dysfunctional or indeed the ligand may not be readily available within the RA synovium [63, 64]. A recent study by Sugiura et al. elegantly demonstrated that high expression of CD80 on DC can restrict the PD-1 pathway during T cell activation [74]. The binding of CD80 to PD-L1 in *cis* on DC can subsequently interfere with the ability of PD-L1 to access PD-1 on T cells. Given that high levels of CD80 on DC has previously been reported within the IA synovium, one could hypothesize that a CD80:PD-L1 interaction similar to that reported by Sugiura et al. could inhibit the PD-1 pathway in IA [75]. Taken together, we have depicted the potential contributions that the PD-1:PD-L1 axis may have in the pathogenesis of joint inflammation in IA in Fig. 2. As highlighted above, previous studies have demonstrated a potential role for PD-1 signalling in cellular bioenergetics, however, limited data exists on the bioenergetic requirements of PD-1^+^ cells in IA. Given that PD-1^+^ cells in the synovium are resistant to PD-1 mediated suppression and do not appear to have an exhausted phenotype, it is tempting to hypothesize that the metabolic requirements of these cells may be aberrant to the metabolic profiles previously reported in infection models. Indeed, Petrelli et al. examined the metabolism of PD-1^+^ and PD-1^−^ CD8^+^ T cells in synovial fluid and demonstrated that PD-1^+^ T cells had increased rates of glycolysis compared to PD-1^−^ T cells [76].

### Immune- related adverse events

To date, antibodies to therapeutically target the PD-1:PD-L1axis have been approved for the treatment of a variety of cancers in the metastatic and adjuvant setting, including melanoma, non-small cell lung cancer (NSCLC), renal cell carcinoma (RCC), Hodgkin’s lymphoma, bladder cancer, head and neck squamous cell carcinoma (HNSCC), Merkel-cell carcinoma, and microsatellite instable-high (MSI-H) or mismatch repair-deficient (dMMR) solid tumours. There are currently five FDA-approved immune checkpoint inhibitors targeting PD-1 (nivolumab, pembrolizumab), or PD-L1 (atezolizumab, durvalumab and avelumab). ICIs such as these have revolutionised the treatment of cancers by significantly improving disease-free survival (DFS) and progression-free duration. However, a caveat of ICI therapy and boosting anti-tumour immune responses is the development of self-reactive T cells which can lead to the induction of various autoimmune or inflammatory diseases, referred to as immune- related adverse events (irAEs). IrAEs are distinct from chemotherapy induced side effects and can persist even after ICI cessation [77]. One such irAE which is becoming increasingly reported, is Inflammatory Arthritis. While exploration into this area is increasing, long term studies and large study cohorts are limited. Indeed, the first description of RA occurring after ICI treatment was in 2017, highlighting the novelty of this research area [78]. In addition to IA, sicca syndrome, myositis, vasculitis and polymyalgia rheumatica represent additional rheumatological irAEs, however IA irAEs appear to be the most common [79–81]. One study reported the development of arthralgia in 13.3% of patients treated with pembrolizumab or nivolumab for metastatic cutaneous malignancies [82], while others prospectively demonstrated the development of IA in 3.8% of patients treated with anti-PD-1: PD-L1 antibodies [83]. Although other irAEs such as colitis, pneumonitis and hypophysitis can develop early during ICI treatment, ICI induced IA possibly develops later in the course of immunotherapy and presents initially with small joint involvement similar to RA [84]. One study reported the manifestation of IA within 6–24 months following commencement of ICI therapy [85]. In those patients who go on to develop IA as a result of ICI treatment, Braaten et al. report that even after cessation of immunotherapy, IA persists [77]. Moreover, this persistent arthritis was less likely to improve in patients with longer ICI treatment duration and in those receiving combination ICI therapy (anti-CTL4 and anti-PD-1). Interestingly, reports of rheumatic irAEs in patients receiving anti-CTLA-4 immunotherapy are rare, suggestive of a more potent role for PD-1 in rheumatology related irAEs [86]. Treatment of IA in this unique cohort of patients may prove challenging given the need to administer immunosuppressive drugs to patients who have previously received immunostimulatory treatments. Hydroxychloroquine (HCQ) was examined as a first line DMARD in a single centre retrospective observational study. The authors used HCQ as a first-line steroid-sparing agent and reported improvements in IA symptoms within their small sample size [87]. In another small case series, Kim et al. report a significant improvement in ICI induced polyarthritis symptoms in three patients in response to tocilizumab [88]. An important unanswered question remains whether use of these immunosuppressive drugs can affect tumour progression. However, early reports appear to be promising. Cappelli et al. demonstrated in a cohort of 60 patients with rheumatological irAEs, that there was no statistically significant increased risk of tumour progression following immunosuppressive treatment. The authors reassuringly reported no change in tumour response in patients treated with either DMARDs or TNF inhibitors [77]. In addition to ICI induced IA, patients may also concurrently develop additional non–rheumatological related irAEs. Cappelli et al. demonstrated within a cohort of 30 ICI induced IA patients, 31% also developed colitis, while thyroid disease, pneumonitis and skin rash were also described within their cohort. These studies demonstrate how rheumatological irAEs can be successfully managed in cancer patients. However, future work should aim to examine therapeutic approaches that will minimize the risk of even developing irAEs in cancer patients undergoing anti-PD-1 treatment. While research into this area is limited, a recent encouraging study by Perez-Ruiz et al. suggests that prophylactic biologic treatment in patients receiving PD-1 immunotherapy may minimise the risk of developing irAEs. Specifically, the authors demonstrated that prophylactic blockade of TNFα before commencing anti-PD-1 (and anti-CTLA4) therapy prevents irAEs (specifically colitis) in mouse models. Importantly, the authors also report that addition of anti-TNFα therapy may also enhance the anti-tumour effects of the PD-1 treatment [89].

## Conclusion

PD-1 is a pleiotropic molecule with a wider than initially described cellular distribution and function. While certain facets of the role of PD-1 in fine-tuning T cell responses have been studied extensively, there is a recent growing appreciation of the role of PD-1 in metabolism and cancer. Multiple studies have confirmed both the expression of PD-1 within the IA synovium and its correlation with disease activity or synovitis. It is therefore logical to assume that in the context of chronic inflammation in the joint, the inhibitory role of PD-1 on T cell function is dysregulated or indeed absent. Furthermore, the emergence of IA irAEs in patients receiving PD-1:PD-L1 immunotherapy underscores the role diminished PD-1 signalling may have in the pathogenesis of IA. Importantly, the function of PD-1 in IA may be unique to other coinhibitory receptors or checkpoint inhibitors given that rheumatic irAEs have not been reported in patients receiving anti-CTLA-4 immunotherapy. Future studies should aim to delineate the function of PD-1 within the IA synovium to better understand how IA irAEs develop and can subsequently be prevented or treated.

## Data Availability

Not applicable.

## Abbreviations

PD-1: Programmed cell death protein 1
irAEs: Immune- related adverse events
IA: Inflammatory Arthritis
APC: Antigen presenting cell
ICI: Immune checkpoint inhibitor
TCR: T cell receptor
NFAT1c: Nuclear factor of activated T cells 1c
TAM: Tumour associated macrophages
BCR: B cell receptor
FAO: Fatty acid oxidation
ECAR: Extracellular acidification rate
OCR: Oxygen consumption rate
SRC: Spare respiratory capacity
LCMV: Lymphocytic choriomeningitis virus
EV: Extracellular vesicles
CIA: Collagen induced arthritis
DC: Dendritic cell
NSCLC: Non-small cell lung cancer
RCC: Renal cell carcinoma
HNSCC: Head and neck squamous cell carcinoma
MSI-H: Microsatellite instable-high
DFS: Disease free survival
NFKB: Nuclear Factor kappa-light-chain-enhancer of activated B cells
HIF: Hypoxia inducible factor
STAT: Signal Transducer and Activator of Transcription.
ITIM: Immunoreceptor tyrosine-based inhibitory motif
ITSM: Immunoreceptor tyrosine-based switch motif
FOXO1: Forkhead Box O1
CTLA: Cytotoxic T-lymphocyte-associated protein
ACPA: Anti–citrullinated protein antibody
DMARD: Disease-modifying antirheumatic drugs
HCQ: Hydroxychloroquine
